# Description on the prevalence of *Proteus mirabilis* through an integrated sampling framework for health, food, and environment in Northeast India and an integrative review with reference to one health context

**DOI:** 10.3389/fmicb.2025.1708233

**Published:** 2026-01-05

**Authors:** Goutam Chowdhury, Tapan Majumdar, Dilem Modi, Karma G. Dolma, Suranjana Chaliha Hazarika, Pallab Sarmah, Samaresh Das, Asish K. Mukhopadhyay, Anup Kumar Ojha, Madhuchhanda Das, Thandavarayan Ramamurthy

**Affiliations:** 1 Division of Bacteriology, ICMR-National Institute for Research in Bacterial Infection, Kolkata, West Bengal, India; 2 Department of Microbiology, Agartala Government Medical College, Agartala, Tripura, India; 3 Bankin Pertin General Hospital and Research Institute, Pasighat, Arunachal Pradesh, India, India; 4 Department of Microbiology, Sikkim Manipal Institute of Medical Sciences, Sikkim Manipal University, Tadong, Gangtok, Sikkim, India; 5 Gauhati Medical College and Hospital, Guwahati, Assam, India; 6 ICMR-Regional Medical Research Centre, Dibrugarh, Assam, India; 7 Centre for Development of Advanced Computing, Kolkata, West Bengal, India; 8 Division of Development Research, Indian Council of Medical Research, New Delhi, India

**Keywords:** one health, food animals, *Proteus mirabilis*, multidrug resistance, diarrhea, virulence gene, whole genome sequence

## Abstract

**Introduction:**

Foodborne infections caused by different pathogens are a perennial public health problem in India. An uncommon enteric pathogen *Proteus mirabilis* has been frequently isolated from the hospitalized diarrheal cases and different food and environmental samples collected from four states in Northeast India. This study was aimed at characterizing *P. mirabilis* isolates to show its etiological importance in public health and also present a review indicating its global prevalence of antimicrobial resistance and detection virulence genes reported from different sources.

**Methods:**

In this study, we have screened 6,298 diarrheal stools from hospitalized patients, 12,305 market foods, 4,270 state-specific foods, and 2,130 environmental samples. *P. mirabilis* was isolated and identified by routine microbiological methods. Representative isolates were examined for antimicrobial susceptibility, putative virulence-encoding genes by PCR, and whole-genome sequencing (WGS) analysis.

**Results:**

Though the isolation rate of *P. mirabilis* was low in diarrheal cases (0.4%), its prevalence was detected mostly in environmental samples (3.2%). All the *P. mirabilis* isolates from diarrheal stools showed resistance to doxycycline, erythromycin, and tetracycline. The majority of the *P. mirabilis* isolates from market foods were resistant to nalidixic acid (92.6%), erythromycin (81.5%), tetracycline, and doxycycline (77.8% each). Isolates from state-specific foods showed higher resistance to quinolone/fluroquinolones, erythromycin, tetracycline, and doxycycline. Meropenem resistance has also been recorded in isolates from market foods and state-specific foods (7.4% and 37.5%, respectively). Of the 15 *P. mirabilis* isolates tested in the PCR assay, the majority of them were positive for *pmfA* and eight other virulence genes.

**Discussion:**

Although these genes are responsible for urinary tract infections (UTIs), some of them are known to induce diarrhea *in vivo*. WGS analysis has identified SPI-1 for the first time in *P. mirabilis,* with other virulence genes associated with diarrhea. Considering its presence in several sources, strengthening the One Health approach is important to implement strategies in order to control *P. mirabilis*-mediated infections.

## 1 Introduction

The genus *Proteus* includes several species, among which *Proteus mirabilis* is clinically significant. This opportunistic pathogen is well-known to cause urinary tract infections (UTIs) in humans (Facciolà et al., 2022). Furthermore, several recent reports indicate the potential association between *P. mirabilis* and diarrheal disease (Müller, 1989; Kharat et al., 2023; Kiiru et al., 2024). *P. mirabilis* is not typically regarded as a major foodborne pathogen compared to other pathogenic species such as *Salmonella* or *Campylobacter*, but it does have important public health implications, particularly in certain populations and settings, as it can also cause gastrointestinal infections. In the context of foodborne illness, *P. mirabilis* can contaminate improperly handled or undercooked food, especially meat, poultry, and dairy products. Food-producing animals (Guo et al., 2019; Yu et al., 2021; El-Saeed et al., 2024; Ramatla et al., 2024) and fermented foods (Keisam et al., 2019) serve as common sources for the transmission of *P. mirabilis*. Foodborne outbreaks due to *P. mirabilis* contamination have also been reported (Cooper et al., 1971; Gong et al., 2019). Infection caused by *P. mirabilis* in food poisoning cases has several clinical symptoms, including nausea, abdominal pain, and severe diarrhea (Shi et al., 2016). The other important concerns regarding *P. mirabilis* are its potential to develop resistance to multiple antibiotics and its ability to acquire and transfer antibiotic resistance genes, thereby promoting the development of resistant strains. Due to these reasons, public health surveillance and appropriate treatment strategies for *P. mirabilis*-associated infections are vital. Several findings indicate the existence of multidrug resistance (MDR) in *P. mirabilis* isolated from the patients and several food sources (Guo et al., 2019; Facciolà et al., 2022; Kiiru et al., 2024). Although many putative virulence factors have been reported in *P. mirabilis*, the specific pathogenic mechanism causing diarrhea in humans is still unclear. Considering its role in the One Health approach, systematic surveillance of different sources, proper infection control, and prudent use of antibiotics are important in minimizing its impact on public health.

The present study was conducted to characterize *P. mirabilis* isolated from the diarrheal cases and different food and environmental samples collected from four states in Northeast India. PCR and whole-genome sequence (WGS) analysis were performed to investigate the genetic features of *P. mirabilis* isolates with reference to their putative virulence factors and antimicrobial resistance genes (ARGs).

## 2 Materials and methods

### 2.1 Collection and processing of samples

As a part of ICMR sentinel diarrheal surveillance, the present study was conducted from 2021 to 2024 in four states of Northeast India, namely Sikkim (Gangtok), Assam (Dibrugarh and Guwahati), Arunachal Pradesh (Pasighat), and Tripura (Agartala). Considering distinct types of samples within each category, a stratified sampling method was used. This strategy leads to more precise estimates for each subgroup. Patients with symptoms suggestive of diarrheal infection, including abdominal pain and watery/bloody stool, were enrolled in this study after obtaining informed consent. For microbial testing, we have screened 6,298 diarrheal stools from hospitalized patients. The stool samples/rectal swabs collected in Carry Blair medium were transported to the Microbiology Laboratory for processing. In addition, 12,305 market foods, 4,270 state-specific foods, and 2,130 environmental samples were collected from shops and vendors, as well as ready-to-eat foods from restaurants and street vendors. The food items included in this study were selected based on high consumption rates, increased risk of contamination, and the fact that they are consumed either raw or in a semi-cooked state, and local culinary practices for consumption and preservation patterns. Environmental samples include stored water, swabs from cutting surfaces/utensils, and hand-wash samples from food handlers.

### 2.2 Isolation and phenotypic characterization of *Proteus mirabilis*

After collection, the samples were processed within 2–3 h and tested for identification of enteric pathogens using the cultural and molecular identification methods as per standard operating procedures developed for the sentinel diarrheal surveillance (https://www.icmrfoodnet.in/static/assets/files/ICMR_StandardOperatingProcedures.pdf; Accessed on October 29, 2025). Suspected *Proteus* spp. isolates were identified using VITEK^®^2 GN Compact system (bioMérieux, Marcy-l’Étoile, France). A total of 43 randomly selected *P. mirabilis* isolates that cover different sources and states were tested for antimicrobial susceptibility (AST) by the Kirby-Bauer disk diffusion method on Mueller-Hinton agar (Difco). The results were interpreted according to the guidelines set by the CLSI (Clinical and Laboratory Standards Institute) (2022). In the AST, commercially available antibiotic discs were used (BD Co., Sparks, MD, USA) that include ampicillin (AM, 10 μg), azithromycin (AZM, 15 μg), cefotaxime (CTX, 30 μg), ceftazidime (CAZ, 30 μg), ceftriaxone (CRO, 30 μg), chloramphenicol (C, 30 μg), ciprofloxacin (CIP, 5 μg), doxycycline (D, 30 μg), erythromycin (E, 15 μg), gentamicin (GM, 10 μg), levofloxacin (LVX, 5 μg) nalidixic acid (NA, 30 μg), norfloxacin (NOR, 10 μg), meropenem (MEM, 10 μg), ofloxacin (OFX, 5 μg), streptomycin (S, 10 μg), tetracycline (TE, 30 μg), and trimethoprim-sulfamethoxazole (SXT, 1.25 and 23.75 μg). *Escherichia coli* strain ATCC 25922 was used as a quality control strain to validate test accuracy.

### 2.3 PCR-based detection of virulence encoding genes

Fifteen randomly selected *P. mirabilis* isolates from different foods were tested for the major virulence genes encoding extracellular metalloprotease (*zapA*), flagellin (*fliC*), hemolysin A of *Proteus* spp. (*hmpA*), mannose-resistant *Proteus-*like fimbria (*mrpA, mrpH*), *P. mirabilis* fimbriae (*pmfA*), *Proteus* toxic agglutinin (*ptaA*), regulator of swarming behavior (*rsbA, rsbR*), urease enzyme large subunit (*ureC, ureG*), and uroepithelial cell adhesin fimbriae (*uca*), using the published primers and PCR conditions (Pathirana et al., 2018; Mirzaei et al., 2019). Overnight cultures of *P. mirabilis* isolates grown in Luria-Bertani broth (BD Difco, USA) were used in the PCR assay. For each test, a total of 2.5 μl of the washed bacterial cells in sterile phosphate buffer saline solution was used as a DNA template.

### 2.4 Genomic DNA sequencing and data analysis

Eight different isolates of *P. mirabilis* identified from humans, vegetables, raw meat, and state-specific foods were included in the WGS. Genomic DNA was prepared using the QIAamp DNA Mini Kit (Qiagen, Hilton, Germany) following the manufacturer’s guidelines. WGS was performed on the HiSeq 2000 (Illumina, San Diego, CA, United States) platform using a paired-end library with an insert size of 150 bp. The presence of ARGs and virulence encoding genes (VEGs) was detected using the ABRicate V1.0.1 tool.

## 3 Results and discussion

### 3.1 Prevalence of *Proteus mirabilis*

*Proteus mirabilis* is known to cause UTI using several virulence mechanisms. This pathogen has also been associated with diarrhea in humans through the consumption of contaminated foods. *P. mirabilis* are ubiquitously present in animals, foods, and environment, and are, therefore, important under One Health context. In addition to diarrheal stool samples, this study was designed to test food samples from the market and different sources in the environment. In any pathogen-specific surveillance, such triangulation of data provides evidence not only to link human illness to food contamination but also improves public health surveillance and guides food safety interventions. In this study, the isolation rate of *P. mirabilis* remained very low (0.4%) in 6298 clinical samples and was detected only in diarrheal cases from Tripura (1.3%) and Arunachal Pradesh (1.9%) (Table 1). Remarkably, of the 12,305 market samples tested, *P. mirabilis* was found higher in food samples collected from Tripura and Arunachal Pradesh (5.6% and 3.2%, respectively), as compared to the other two states (Table 1). *P. mirabilis* was present in 74 (3.9%) state-specific foods collected from Assam.

**Table 1:** State-wise prevalence of *P. mirabilis* in diarrheal, market food and state-specific food samples.

Type of sample	State/percent positive (No positive/total sample)				Total %
	Arunachal Pradesh	Assam	Sikkim	Tripura	
Diarrheal stool	1.9 (11/569)	(0/1051)	(0/3747)	1.3 (12/931)	0.4 (23/6298)
Market foods	3.2 (39/1206)	0.5 (20/4199)	1.3 (56/4443)	5.6 (139/2457)	2.1 (254/12,305)
State-Specific Foods	0.8 (6/712)	3.9 (74/1894)	0.8 (7/875)	0.8 (6/789)	2.2 (93/4270)
Environmental samples	4.7 (44/926)	(0/178)	0.0 (0/331)	3.7 (26/695)	3.2 (70/2130)

*Proteus mirabilis* was detected in 3.2% (70/2130) of the environmental samples that include cutting surfaces/utensils, had-wash samples from food handlers, and stored water (Table 2). Reports on the prevalence of this pathogen in such environmental samples were not found in the existing literature. Raw/dried meat (111/12,305) showed slightly higher positivity for *P. mirabilis* (Table 3). In several studies, the presence of *P. mirabilis* has been reported more in animal foods (Sanches et al., 2019; Yu et al., 2021; Ma et al., 2022; El-Saeed et al., 2024), aquatic products (Ma et al., 2022), and abattoirs (Bhandare et al., 2010) than in vegetables (Li et al., 2023). Among the state-specific foods, *P. mirabilis* was detected more in fermented small animals (13/185) (Table 4). As reported in other studies, the prevalence of *P. mirabilis* seems high among fermented foods of the Northeast regions in India (Keisam et al., 2019; Kumar et al., 2019; Anand Singh et al., 2023).

**Table 2:** Detection of *P. mirabilis* from the environmental samples.

Source	No. of samples tested	No of samples positive for *P. mirabilis* (%)
Environment (cutting surfaces, hand-washing from food-handlers, utensils)	1,606	62 (3.9)
Stored water	524	8 (1.5)
Total	2,130	70 (3.2)

**Table 3:** Detection of *P. mirabilis* from market samples.

Source	No. of samples tested	No of samples positive for *P. mirabilis* (%)
Ckung	69	3 (4.3)
Khar, Outenga	16	
Dough and batter	316	4 (1.3)
Fermented/processed/preserved	420	3 (0.7)
Fruits, vegetables and salads	2,101	20 (0.9)
Milk products and sweets	302	4 (1.3)
Processed milk products and sweets	873	4 (0.4)
Non-vegetarian foods	1,080	27 (2.5)
Raw/dried meat	2,309	111 (4.8)
Raw/dry fish	2018	43 (2.1)
Refrigerated foods	161	6 (3.7)
Rice, flour, pulses	1,467	19 (1.3)
Kinema	40	
Spices	27	1 (3.7)
Bangoi, godak	9	
Vegetables	1,097	9 (0.8)
Total	12,305	254 (2.1)

**Table 4:** Detection of *P. mirabilis* from state-specific fermented foods.

Source	No. of samples tested	No of samples positive for *P. mirabilis* (%)
Alcoholic Brew	23	
Bamboo Shoots	260	4 (1.5)
Beer	32	
Cereals	716	5 (0.7)
Fish	936	42 (5.9)
Insects	135	8 (5.9)
Meat	147	4 (2.7)
Milk Products	122	
Small animals	185	13 (7.0)
Soyabean	128	1 (0.8)
Vegetables	943	11 (1.2)
Wine	643	5 (0.8)
Total	4,270	93 (2.2)

### 3.2 Virulence encoding genes (VEGs)

The pathogenicity of *P. mirabilis* involves several virulence factors (Chakkour et al., 2024). Of the 15 *P. mirabilis* isolates tested in the PCR assay, 80% of the isolates were positive for *pmfA* (*P. mirabilis* fimbriae), and 73% were positive for *mrpA/mrpH*, *ureC*, *ptaA*, *hmpA*, *zapA*, *fliC*, and *rsbAR* (Table 5). The *uca* and *ureG* genes were detected in 67% and 40% of the isolates, respectively. Most of these genes have been reported in *P. mirabilis* isolated from UTI cases. Several reports indicated that *P. mirabilis* isolates harboring *ireA* (siderophore receptors), *ptaA,* and *zapA*, *ucaA, pmfA*, *atfA* (ambient temperature fimbriae is a type 1 major fimbrial subunit), and *mrpA*, *fliC*, *hlyA* (hemolysin A), and *hpmA* genes are strongly associated with their pathogenesis (Barbour et al., 2012; Sanches et al., 2019; Li et al., 2022; Ramatla et al., 2024).

**Table 5:** PCR assay results of virulence-encoding genes in *P. mirabilis* isolated from different sources.

Sample ID/Source	Center	Virulence gene										
		*mrpA*	*pmfA*	*uca*	*ureG*	*ptaA*	*hmpA*	*zaPA*	*fliC*	*mrpH*	*ureC*	*rsbAR*
ASM/KMR/RAWFISH/4142	Assam											
ASM/KMR/DRYFISH/4141	Assam											
ASM/KMR/COOKEDMEAT/4153	Assam											
ASM/KMR/COOKEDFOOD/4123	Assam											
ASM/KMR/ENVIRONMENT/2961	Assam											
ASM/KMR/COOKEDMEAT/3901	Assam											
ASM/KMR/RAWFISH/2964	Assam											
ASM/KMR/COOKEDMEAT/4355	Assam											
ASM/KMR/VEGETABLE/3221	Assam											
AR/BPGH/MS/2090	Arunachal Pradesh											
ASM/KAM/SSF/S7	Assam											
ASM/KAM/SSF/S6	Assam											
ASM/KAM/SSF/S39	Assam											
ASM/DIB/RAWMEAT/921	Assam											
SKM/SSK/RAWMEAT/2517	Sikkim											

*“Blue,” Positive; “Brown,” Negative.*

In several studies, the presence of virulence factors and VEGs has been reported in *P. mirabilis* isolated from diarrheal patients, fermented foods, food animals, and contaminated foods. *ureC, hamA, zapA,* and *rsmA* (regulation of swarming motility) were identified in diarrheal cases (Table 6). The *virB* gene associated with a type IV secretion system was reported to play a role in causing diarrhea in humans (Shi et al., 2016; Gong et al., 2019). *P. mirabilis* isolated from food poisoning cases demonstrated *in vitro* and *in vivo* gastrointestinal pathogenicity, including adherence and invasiveness (Shi et al., 2016). Furthermore, biofilm production was notably associated with the expression of *ureC, zapA, rsmA, hmpA, mrpA, atfA,* and *pmfA* genes and was also shown to induce diarrhea in mice (Sun et al., 2020).

**Table 6:** Reported virulence factors and virulence encoding genes in *P. mirabilis* isolated from diarrheal patients, fermented foods, food animals, and contaminated foods.

Source	Virulence	Major virulence encoding gene	Reference
Chicken	Siderophore, proteases, fimbriae, hemolysins	*ireA* (siderophore receptors), *ptA,* and *zapA* (proteases), *ucaA, pmfA, atfA*, and *mrpA* (fimbriae), *hlyA* and *hpmA* (haemolysins)	Ramatla et al. (2024)
Fermented foods	Hemolysin, urease	*hpm* and *ure*	Keisam et al. (2019)
Diarrheal patients	Type IV secretion system, hemolysins, metalloprotease	*ureC, rsmA, hamA, zapA*	Shi et al. (2016)
Chicken	Aggregative adherence in HEp-2, biofilm formation, cytotoxicity in Vero cells	*mrpA, ucaA, pmfA, atfA, ireA, ptA, zapA, hpmA*	Sanches et al. (2019)
Broiler farms	Siderophore, proteases, fimbriae	*ireA, hpmA, ucaA, mrpA, atfA, zapA,* and *ptA*	Li et al. (2022)
Animals with diarrhea	Biofilm production	*ureC* (urease)*, zapA, rsmA, hmpA, mrpA, atfA*, and *pmfA*	Sun et al. (2020)
Pig farm	Fimbriae, siderophore, siderophore	*mrp, pbtA, hpm*	Qu et al. (2022)
Chicken	Fimbriae, proteases	*zapA, ucaA, hampA*	Sarwar et al. (2025)

*P. mirabilis* is primarily known for its role in UTI and wound infections, but it has also been implicated in gastrointestinal infections. Unlike *Salmonella* and *Shigella*, the diarrheal pathogenesis of *P. mirabilis* is poorly established. The pathogenesis of diarrheal disease caused by *P. mirabilis* is multifactorial and includes mucosal lining adherence and colonization, biofilm formation in the gut (Palusiak, 2022), production of urease in causing intestinal dysbiosis (Grahl et al., 2023), secretion of hemolysins (Elhoshi et al., 2023), and several other secreted factors leading to inflammation and diarrhea (Shi et al., 2016).

Using WGS, different VEGs have been identified in *P. mirabilis.* However, VEGs specific for the diarrheal infection, which are present in the other enteric pathogens, have not been reported in *P. mirabilis*. In the WGS analysis, we identified potential VEGs associated with diarrheal infections (Table 7). SPI-1 (*Salmonella* Pathogenicity Island 1) is a gene cluster primarily associated with *Salmonella* species that enables them to invade host epithelial cells. SPI-1 encodes a Type III Secretion System (T3SS) which injects effector proteins into host cells, triggering rearrangements of the host cell’s cytoskeleton and allowing bacterial internalization. The majority of *Salmonella* virulence genes are located within these distinct genomic regions of SPIs, which have been acquired by independent horizontal transfer events (Marcus et al., 2000; Galán, 2001). SPI-1 is composed of 39 genes, including *inv*, *hil*, *prg*, *org*, *spa*, and *sip/ssp* genes, and plays a key role in *Salmonella* pathogenesis (Boyd et al., 1997). The majority of the known invasion genes are located between centisomes 62 and 64 of the chromosome, and SPI-1 is located at centisome 63 (Hansen-Wester and Hensel, 2001). Several SPI-1 gene components including *invC, invG, spaP,* and *SpaQ* have been identified in *P. mirabilis* isolates, which is a significant finding. To the best of our knowledge, this is the first report of SPI-1 in *P. mirabilis* isolates. Our work is in progress to map the entire SPI-1 gene cluster of *P. mirabilis* using the WGS data.

**Table 7:** Whole genome sequence analysis of *P. mirabilis* isolates harboring different antimicrobial resistance and virulence encoding genes.

ICMR FoodNet ID	Center	Resistance genes	Virulence genes
TRP/DHL/HUMAN/1/1	Tripura	Chloramphenicol: *catA1, catA4* Aminoglycoside: *aac(3)-Iid, aadA5, aadA, aph(6)-Id, aph(3′)-Ia* Sulfonamide: *sul1, sul2* Beta-lactamase: *bla*_TEM-1_ Trimethoprim: *dfrA1, dfrA17* Antibiotic efflux: *qacEΔ1*	TTSS (SPI-1 encode): *invC, invG, spaP, spaQ* Stress adaptation: *sodCI* Adherence: *papC, fimB, fimA*, Endotoxin: *htrB, lpxH, lpxK*, Invasion: *cheR, cheZ, motA*
TRP/DHL/HUMAN/2/1	Tripura	Chloramphenicol: *floR, catA4* Aminoglycoside: *aadA2, aph(6)-Id, aph(3″)-Ib, aph(3′)-Ia* Sulfonamide: *sul2* Macrolide: *ereA* Trimethoprim: *dfrA32*	TTSS (SPI-1 encode): *invC, invG, spaP, spaQ* Stress adaptation: *sodCI, katA* Adherence: *papC, fimB, fimA*, Endotoxin: *htrB, lpxH, lpxK*, Invasion: *cheR, cheZ, motA*
TRP/DHL/HUMAN/4/1	Tripura	Chloramphenicol: *catA4*	TTSS (SPI-1 encode): *invC, invG, spaP, spaQ* Stress adaptation: *sodCI, katA* Adherence: *papC, fimB, fimA*, Endotoxin: *htrB, lpxH, lpxK*, Invasion: *cheR, cheZ, motA*
TRP/DHL/HUMAN/5/1	Tripura	Chloramphenicol: *catA4*	TTSS (SPI-1 encode): *invC, invG, spaP, spaQ* Stress adaptation: *sodCI, katA* Adherence: *papC, fimB, fimA*, Endotoxin: *htrB, lpxH, lpxK*, Invasion: *cheR, cheZ, motA*
TRP/DHL/HUMAN/6/1	Tripura	Chloramphenicol: *catB3, catA4, cmlA1* Aminoglycoside: *aadA, aac(6′)-Ib10, aph(3″)-Ib, aph(6)-Id,* *aph(3′)-Ia* Sulfonamide: *sul3, sul1, sul2* Beta-lactamase: *bla*_TEM-1_, *bla*_CTX-M-3_, *bla*_OXA-1_ Tetracycline: *tet(A)* Trimethoprim: *dfrA1* Antibiotic efflux: *qacEΔ1*	TTSS (SPI-1 encode): *invC, invG, spaP, spaQ* Stress adaptation: *sodCI, katA* Adherence: *papC, fimB, fimA*, Endotoxin: *htrB, lpxH, lpxK*, Invasion: *cheR, cheZ, motA*
ASM/KAM/VEGETABLE/ 1,204	Assam	Chloramphenicol: *catA4*	TTSS (SPI-1 encode): *invC, invG, spaP, spaQ* Stress adaptation: *sodCI, katA* Adherence: *papC, fimB, fimA*, Endotoxin: *htrB, lpxH, lpxK*, Invasion: *cheR, cheZ, motA*
SKM/SSK/RAWMEAT/4248	Sikkim	Chloramphenicol: *catA4*	TTSS (SPI-1 encode): *invC, invG, spaP, spaQ* Stress adaptation: *sodCI, katA* Adherence: *papC, fimB, fimA*, Endotoxin: *htrB, lpxH, lpxK*, Invasion: *cheR, cheZ, motA*
ASM/KAM/SSF/S39	Assam	Chloramphenicol: *catA4*	TTSS (SPI-1 encode): *invC, invG, spaP, spaQ* Stress adaptation: *sodCI, katA* Adherence: *papC, fimB, fimA*, Endotoxin: *htrB, lpxH, lpxK*, Invasion: *cheR, cheZ, motA*

A growing body of evidence suggests that these putative VEGs have been identified in several Gram-negative pathogens and are linked with diarrheal infection. In *Salmonella enterica*, the surface-presenting antigen *inv-spa* complex has been reported as a potential virulence factor (Boyd et al., 1997). The adherence*-*linked *pap* (pappyelonephritis-associated pilus) is important in P fimbriae assembly in *Escherichia coli* and also responsible for causing diarrhea in young animals (Ngeleka et al., 2019; Renzhammer et al., 2020). Lipid A 4′-kinase encoded in the gene *ipx* acts as an endotoxin, which is expressed in many Gram-negative infections, including septic shock (Garrett et al., 1997; Raetz and Whitfield, 2002). *ipx* and the lipid-A biosynthesis gene *htrB* are considered potential virulence factors in *Salmonella* spp. (Jones et al., 1997) and *Campylobacter* spp., (Stoakes et al., 2023), respectively. In *Campylobacter* spp., *Salmonella* spp., and *E. coli*, the genes *cheR, cheZ* and *motA* regulate adhesion, motility ability, invasion and biofilm formation (Vilas Boas et al., 2024; Koolman et al., 2015). Identification of these VEGs underscores the pathogenic potential of *P. mirabilis.* However, the role of virulence genes in causing diarrheal infection needs further investigation. Several WGS analyses of *P. mirabilis* have been made using isolates from humans (Ghiglione et al., 2025; Zamudio et al., 2025), foods (Ma et al., 2022; Tan et al., 2025), and animals/birds (Khudeir et al., 2023; Ma et al., 2023; Su et al., 2025). Some of these studies compared genomic consistency, VEGs, AMR genes, and lineage analysis.

### 3.3 Antimicrobial resistance in *Proteus mirabilis*

**Figure 1:**
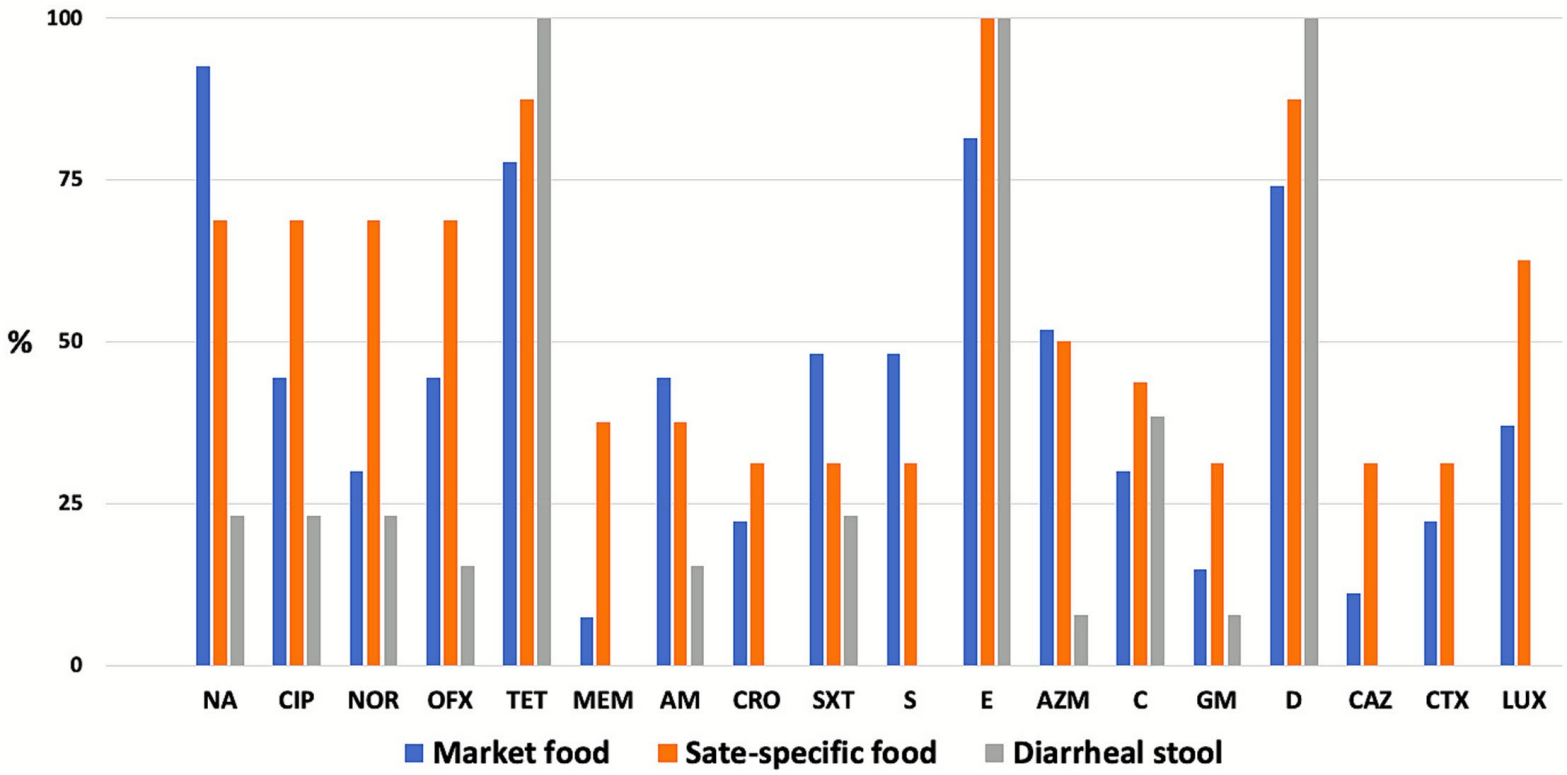
Antimicrobial resistance patterns of *P. mirabilis* isolated from different sources (*n* = 43). AM, ampicillin; AZM, azithromycin; CTX, cefotaxime; CAZ, ceftazidime; CRO, ceftriaxone; C, chloramphenicol; CIP, ciprofloxacin; D, doxycycline; E, erythromycin; GM, gentamicin; LVX, levofloxacin; NA, nalidixic acid; NOR, norfloxacin; MEM, meropenem; OFX, ofloxacin; S, streptomycin; TE, tetracycline; and SXT, trimethoprim-sulfamethoxazole.

All the tested *P. mirabilis* isolates from diarrheal stool samples showed resistance to doxycycline, erythromycin, and tetracycline (Figure 1). However, these isolates were susceptible to tetracycline, ceftazidime, ceftriaxone, and levofloxacin. From market samples, majority of the *P. mirabilis* isolates are resistant to nalidixic acid (92.6%), erythromycin (81.5%), tetracycline (77.8%), doxycycline (77.8%). Only two isolates showed resistance to meropenem (7.4%; Figure 1). State-specific foods, mostly locally prepared fermented foods, showed a higher prevalence of AMR *P. mirabilis* than the market food isolates. Higher resistance (>50%) has been seen with quinolone/fluroquinolones, erythromycin, tetracycline, and doxycycline. Meropenem resistance has also been recorded in isolates from market foods and state-specific foods (7.4% and 37.5%, respectively).

The majority of the clinical isolates were multidrug resistant (MDR). High resistance to quinolone/fluroquinolones, tetracycline has been reported in *P. mirabilis* isolated from chickens (Ramatla et al., 2024; Sarıçam İnce and Akan, 2024), cotrimoxazole (59.32%) from dairy products (Lordejani et al., 2025), doxycycline, tetracycline, sulfamethoxazole, kanamycin, and cephalothin from diarrhetic animals (Sun et al., 2020), carbapenem resistance from clinical sources (Facciolà et al., 2022; ElTaweel et al., 2024).

We have tested five representative *P. mirabilis* strains in the WGS analysis. This analysis has identified several ARG alleles such as aminoglycoside acetyltransferase (*aadA2, aadA5*) aminoglycoside phosphotransferase [*aac(3′)-lid, aph(3′)-Ia, aph(3′)-Ib, aph(6′)-Ib10, aph(6′)-Id*], beta-lactamase (*bla*_TEM-1,_ *bla*_CTX-M-3,_ *bla*_OXA0-1_), chloramphenicol acetyltransferase (*catA1, catA4, catB3*, *cmlA*) dihydrofolate reductase (*dfrA1, dfrA17, dfrA32*), erythromycin esterase (*ereA*) florfenicol resistance (*floR*), and sulfamethoxazole resistance (*sul1, sul2, sul3*). Two isolates from diarrheal cases and three food isolates harbored *catA4*, which encodes resistance to chloramphenicol (Table 7).

Several studies indicate that *P. mirabilis* isolated from diarrheal patients, food animals, and contaminated foods is MDR (Table 8). ARGs identified from animal foods include *aad, aac, aph, bla*_CTX,_ *bla*_OXA,_ *cat, dfr,* and *sul*. The self-transmissible mobile genetic element SXT/R391 is an integrative and conjugative element (ICE). Interestingly, *P. mirabilis* isolates from the non-human sources harbored ICE*Pmi*ChnHBRJC2, ICE*Pmi*ChnSC1111, ICE*Pmi*ChnJZ26, ICE*Pmi*ChnChSC1905, ICE*Pmi*ChnS012, and ICE*Pmi*Jpn1 (Table 8). The SXT/R391 ICEs carry the ARGs encoding resistance to chloramphenicol, kanamycin, streptomycin, trimethoprim-sulfamethoxazole, and tetracycline (Li et al., 2016). In addition to ICEs, *P. mirabilis* can easily acquire and transfer ARGs to other bacterial species through several mobile genetic elements such as plasmids, insertion sequences, and transposons (Girlich et al., 2020). *Salmonella* genomic island-1 (SGI1) is an integrative mobile element originally identified in the MDR *Salmonella* Typhimurium DT104 clone (Boyd et al., 2001). SGI1 and *Proteus* genomic island (PGI) in *P. mirabilis* have been found to carry several important ARGs (Boyd et al., 2001; Wang et al., 2019). *P. mirabilis* isolates are mostly susceptible to fluoroquinolones (Wang et al., 2019). Some of the isolates from chicken sources carried plasmid-mediated *qnrD, qnrA1, and qnrS1* genes, which are shown to express resistance to fluoroquinolones (Table 8).

**Table 8:** Reported antimicrobial resistance and AEGs of *P. mirabilis* isolated from the food animals/contaminated foods.

Source	Antimicrobial resistance	Predominant AMR encoding gene	Reference
Meat and aquatic products	Trimethoprim-sulfamethoxazole, streptomycin, chloramphenicol, ampicillin, nalidixic acid, norfloxacin, florfenicol, gentamicin, fosfomycin	ICE*Pmi*ChnJZ26 *aac(6′)-Ib-cr*, *bla*_CTX-M_, *fosA3*	Ma et al. (2022)
Chicken	Ampicillin, ceftiofur, amoxicillin-clavulanic acid, cephalotin, cefoxitin, ceftazidime, ceftriaxone, cefotaxime, cefepime, nalidixic acid, norfloxacin, enrofloxacin, ciprofloxacin, trimethoprim-sulfamethoxazole, aztreonam, chloramphenicol, gentamicin	*bla*_CTX-M-2_, *qnrD*	Sanches et al. (2019)
Broiler farms	Chloramphenicol, ciprofloxacin, trimethoprim-sulfamethoxazole, imipenem, and meropenem	*stcM, aac(6′)-Ib-cr, cmlA, bla*_CTX-M_ *bla*_OXA_*, qnrA, bla*_NDM-1_	Li et al. (2022)
Food-producing animals	Ampicillin, amoxicillin-clavulanic acid, cefotaxime, ceftriaxone, chloramphenicol, florfenicol, nalidixic acid, ciprofloxacin, streptomycin, spectinomycin, apramycin, doxycycline, trimethoprim-sulfamethoxazole	*Salmonella* genomic island 1 (SGI1) *aadA2, aac(6′)-Ib-cr, bla_OXA-1_, catB3, arr-3*	Wang et al. (2019)
Pig farms	tetracyclines, macrolides, sulfonamides, *β*-lactams, and chloramphenicol	*rmtB, sul1, qnrS1, aac(6′)-Ib-cr, bla*_CTX-M-65,_ *bla*_OXA-1_	Qu et al. (2022)
Chicken	Beta-lactams, quinolones, sulfonamides	*bla*_CTX-M_, *bla*_EM_, *bla*_OXA_, *bla*_CMY,_ *qnrA, qnrD, qnrS, sul1, sul2*	Sarwar et al. (2025)
Clinical and foods	Tetracycline, minocycline, doxycycline, sulfisoxazole, streptomycin, gentamicin, colistin, and polymyxin B	SGI1 and *Proteus* genomic island (PGI) *cmlA5*, *dfrA14*, *bla*_OXA-1_, *aadA15*, *bla*_OXA-10_, *catB3, dfrA16*	Xiao et al. (2019)
Farm animals	sulfamethoxazole, ampicillin, florfenicol, ciprofloxacin, amoxycillin/clavulanic acid, cefatriaxone, chloramphenicol, levofloxacin, gentamicin, Fosfomycin, tetracycline	SXT/R391 ICEs ICE*Pmi*ChnHBRJC2, *erm* ICE*Pmi*ChnSC1111, *tet*, *bla*_CTX M-65_, *aac(6′)-Ib-cr*	Han et al. (2024)
Swine	trimethoprim-sulfamethoxazole, rifampicin, tetracycline, chloramphenicol, fosfomycin	ICE*Pmi*ChnChSC1905 *aac(3)-IV, aadA1, aadA2b, aph(3″)-Ib, aph(3′)-Ia, aph(4)-Ia*, *aph(6)-Id* *aac(6′)-Ib-cr, bla*_CTX-M-65_, *bla*_OXA-1,_ *fosA3, ere(A), cat, catB3*, *floR, arr3, sul1*, *sul2, tet(C)*, *tet(J), dfrA1*, *dfrA32*	Song et al. (2022)
Chicken and swine	Amoxicillin-clavulanate, ampicillin, aztreonam, ceftazidime, chloramphenicol, ciprofloxacin, ceftriaxone, cefotaxime, florfenicol, fosfomycin, nalidixic acid, norfloxacin, spectinomycin; streptomycin, trimethoprim-sulfamethoxazole	*qnrA1*, *lun*, *dfrA1, aadA1, aac(6′)-Ib-cr, bla*_DHA_, *bla*_PER-12_, *bla*_PER-16_	Chen et al. (2022)
Chicken	Spectinomycin, fosfomycin, cephalosporins, aztreonam, erythromycin, kanamycin, and gentamicin	ICE*Pmi*ChnS012 *aac(6′)-Ib-cr, fosA3*, *bla*_OXA-1_, *bla*_CTX-M-65_, *aph3-VI*, *bla*_HMS-1_ *dfrA1, ereA1, aadA2*	Lu et al. (2021)
Food animals	ceftazidime, cefotaxime, ceftriaxone	*bla*_TEM_, *bla*_OXA_, *bla*_SHV_, *bla*_FOX_, *bla*_CIT_, *bla*_CTX-M1_, *bla*_CTX-M9_, *bla*_CTX-M2_, *bla*_DHA_, *bla*_EBC_	Chinnam et al. (2021)
Chicken	Chloramphenicol, trimethoprim-sulfamethoxazole, streptomycin, spectinomycin, cephalosporins, aztreonam, fosfomycin, kanamycin, and gentamicin	ICE*Pmi*Jpn1 *bla*_CTX-M-3_, *fosA3* *dfrA16*, *bla*_CARB-2_, *aadA2, cmlA1*, *aadA1* *catA1, bla*_TEM-1b_, *aphA1a, sul2, strA* and *strB*	Lei et al. (2020)
Chicken	Chloramphenicol and sulfonamides	*floR*, *sul1*	Soliman et al. (2018)

The pathogenesis of diarrheal disease caused by *P. mirabilis* is complex and involves multiple mechanisms, including adherence to intestinal surfaces, urease production, toxin secretion, and induction of inflammation. Although *P. mirabilis* is not among the most common causes of gastroenteritis, its ability to disrupt gut homeostasis and to express a repertoire of virulence factors makes it a potential contributor to gastrointestinal diseases.

## 4 Limitations of this study

There are a few limitations in this study that could be addressed in future research. Although we collected a large number of samples from different sources, statistical analysis was not done because of the high variability in the prevalence of *P. mirabilis*. WGS data are limited to a few isolates, and large-scale analyses must include identification of sequence types (ST) through core genome multilocus sequence typing (MLST), identification of SPI-1 regulatory genes, and pangenome analysis. Our findings suggest the presence of a *Salmonella*-like type III secretion module in the genomes of *P. mirabilis*. However, this study does not present the structural organization of these genes or the genomic island context that would firmly establish an SPI-1-like cluster in *P. mirabilis*. No *in vivo* study has been conducted to confirm the functional or virulence expression associated with gastrointestinal pathogenicity.

## 5 Contribution and implications

This study demonstrates the prevalence of *P. mirabilis* in diarrheal patients and in food and environmental samples collected in NE India. Market- and state-specific food items showed greater positivity toward this pathogen. The identified *P. mirabilis* isolates from several sources harbored virulence genes not only responsible for UTI but also capable of causing diarrheal infection. Overall, our findings emphasized the need to include *P. mirabilis* in routine surveillance as an indicator of food quality. Effective control and prevention strategies for *P. mirabilis*-associated infections rely on a combination of hygiene practices, antibiotic stewardship, and monitoring in high-risk settings like healthcare facilities and food processing plants. Regular surveillance of healthcare-associated infections can help identify contamination load and outbreaks of *P. mirabilis*.

## 6 Conclusion

While *P. mirabilis* may not be as frequently implicated in foodborne illness as other pathogens, it remains a public health concern due to its potential to cause opportunistic infections. Its role in healthcare-associated infections and the growing issue of antimicrobial resistance. The prevalence of *P. mirabilis* isolated from several foods from Northeast India is a major concern. Reports from other studies also support the potential of *P. mirabilis* as a foodborne pathogen. *P. mirabilis* isolated in this study harbored several VEGs and ARGs and hence may have an association with food contamination/diarrheal disease. However, further detailed studies are warranted to strengthen the hypothesis with well-designed *in vitro* and *in vivo* investigations. To control infections caused by *P. mirabilis*, several public health measures, such as proper sanitation, adequate surveillance, monitoring, and tracking AMR trends, need to be addressed under the One Health approach. Understanding the routes of transmission of *P. mirabilis* and adhering to best practices in food safety and infection control are key in mitigating its impact.

## Data Availability

The raw data supporting the conclusions of this article will be made available by the authors, without undue reservation.
